# Supplementary material for: Are carbon emissions trading and green financial instruments synergistic? -Comprehensive quantitative research based on content analysis
Source: PLoS One. 2024 Mar 7;19(3):e0298601. doi: 10.1371/journal.pone.0298601 (PMC10919601; doi:10.1371/journal.pone.0298601)
Supplement: S1 Data — (ZIP) [file pone.0298601.s001.zip › Data/地级市Z_F工作报告.zip!/╣π╬≈/╣≡┴╓╩╨/└┤╫╘╣1⁄2╓┌║┼í╛┬φ┐╦╩2╛▌═°í┐_2014.docx]

各位代表：

　　现在，我代表市人民政府向大会作工作报告，请予审议，并请各位政协委员和列席会议的同志提出意见。

　　一、2013年工作回顾

　　过去的一年，面对错综复杂的形势，我们在自治区党委、政府和市委的领导下，按照“加快建设新城，着力提升老城，产业融合发展，城乡协调推进，生态文化相融，富裕和谐桂林”的总要求，牢牢把握主题主线，落实改革创新措施，增强发展动力活力，抓紧抓实旅游胜地建设等事关全局的重大工作，促进经济增长、效益提升、结构优化、民生改善、社会和谐。初步核算，地区生产总值1657.90亿元，增长11%；组织财政收入180.37亿元，增长10.3%；全社会固定资产投资1390.32亿元，增长25.4%；规模以上工业增加值601.99亿元，增长18.8%；社会消费品零售总额604.03亿元，增长12.6%；城镇居民人均可支配收入24552元，农民人均纯收入8361元，分别增长10.1%、14.1%；居民消费价格涨幅2.5%。

　　(一)着力推动规划纲要落实，旅游胜地建设实现“开门红”。以落实《桂林国际旅游胜地建设发展规划纲要》为抓手，精心组织，周密部署，综合施策，旅游胜地建设精彩开局，桂林旅游升级版加快打造。政策落地取得实质进展。临桂撤县设区、《桂林漓江风景名胜区总体规划》、《桂林旅游产业用地改革试点总体方案》、智慧城市试点获国家层面批准，外国游客过境72小时免签、入境停留144小时免签已获国家有关部门同意，专项转移支付、境外旅客购物离境退税等一批政策获国家有关部门积极回应和支持。全区旅游发展大会在我市召开，建设桂林国际旅游胜地成为全区共识。宣传招商互动推进。先后赴北京、香港、深圳、南宁、台北举办新闻发布会和项目推介会，通过媒体在海内外刮起了“桂林旋风”，桂林知名度、美誉度和投资认可度持续提升。新签市外境内项目总投资750亿元；内资区外到位资金572.11亿元，增长10.5%；实际利用外资3.9亿美元，增长11.7%。招大引强创历史新高，万达、华润、保利、海吉星、港中旅、海航、中电科等大型优质企业落户桂林。项目带动成效显著。以旅游胜地十大重中之重项目为引领，实现了项目引进快、落地快、开工快、建设快的“桂林速度”。全年实施重大项目708项，完成投资628亿元，78个项目竣工投产。“北通南畅”工程扎实推进，两江国际机场航站楼及站坪配套设施扩建项目启动，保利文化产业园项目开工建设，万达文化旅游产业城和万达广场项目、海吉星食尚港项目成功签约，漓江保护利用和经营管理改革进展顺利，中电科光通信产业园、华润万象城及五彩城大型商业综合体、体育休闲产业等项目前期工作加快推进。法国地中海俱乐部桂林愚自乐园度假村投入运营，金世邦国际足球旅游文化产业园、罗山湖体育运动旅游休闲项目、玉圭园·环球名胜等高端旅游产品加快建设。旅游品质持续提升。坚持大旅游、大产业、大发展的理念，着力推动旅游高端化、精品化、差异化发展。开展第二期中国南方喀斯特世界自然遗产申报。成功举办第七届联合国世界旅游组织/亚太旅游协会旅游趋势与展望国际论坛、第四届桂林国际山水文化旅游节、第四届中国桂林国际旅游博览会、第五届中国桂林创新创意文化节暨国际动漫节，品牌展会影响力持续提升。深入贯彻《中华人民共和国旅游法》，旅游市场监管和旅游诚信体系建设进一步完善。全市接待旅游总人数3584万人次，其中入境游客194万人次，分别增长8.9%、6.2%；实现旅游总收入348.48亿元，其中入境旅游收入53.85亿元，分别增长25.9%、16.1%。我市荣获“中国十大休闲城市”“中国十佳品牌会展城市”，跻身全国旅客满意度十强，漓江入选美国CNN网评全球15条最美河流。

　　(二)着力调结构促转型，产业优化升级取得新进展。强力推动新型工业化、农业现代化与现代服务业融合发展，经济总量增大，发展势头增强。工业经济提质增效。全市规模工业总产值1917.11亿元，增长20.1%，实现利税237.4亿元，工业经济效益综合指数提高7.5个百分点。君泰福电气“绿色能源·智能电网产业园”等21个重大工业项目竣工投产。电子信息、医药及生物制品、先进装备制造、生态食品四大优势产业占工业总产值的比重达47.5%，提升2个百分点；新能源、新材料等战略性新兴产业产值突破450亿元，增长20%。其中，医药及生物制品等3个产业成长为百亿元产业，全市超百亿元产业达到9个；风能等新能源建设项目数占全区半壁江山。园区集聚功能增强，高新区、苏桥经济开发区、西城经济开发区工业总产值分别达275亿元、102亿元、302亿元，分别增长18%、24%、17%；荔浦、兴安工业集中区产值超100亿元，灵川、平乐、恭城、灌阳工业集中区产值超50亿元。桂林“两化”融合试验区建设顺利通过国家验收。桂林力源集团成为我市首个产值超百亿元企业。农业农村稳步发展。完成农林牧渔业总产值471.74亿元，实现增加值299.44亿元，分别增长5.5%、5.2%。粮食总产量突破200万吨，荣获全区粮食生产先进市；水果、蔬菜、食用菌等特色效益经济作物总产量位居全区前列；肉类、水产品总产量持续增长。农业农村基础进一步夯实。桂林防洪及漓江补水枢纽工程川江水库基本建成、小溶江水库大坝封顶、斧子口水库实现截流，实施153座中小型病险水库除险加固工程，新增、恢复和改善灌溉面积35.19万亩，解决37.02万农村人口饮水安全问题；新农村建设扎实推进，新建“普惠制”新村400个，累计建成1681个；新增通水泥路建制村144个，通畅率达76.8%；完成1531个自然村(屯)内道路硬化，累计完成11599个，占20户以上自然村的81.6%；强农惠农富农政策全面落实，累计发放农机购置、农资综合及良种补贴资金5亿多元。现代服务业活力趋强。启动服务业标准化建设，建立服务业引导基金，积极推动金融、物流、信息、健康养生等产业发展。实施服务业重点项目226项；服务业实现增加值565.59亿元，增长7.3%，对全市税收贡献率达62%。辖区银行业金融机构本外币存款余额突破2000亿元大关、贷款余额1225亿元，分别增长13%和16.4%。两江国际机场旅客年吞吐量587.5万人次，国际直航旅客吞吐量保持全区第一。中国(桂林)国际健康养生服务产业创新发展高端论坛成功举办。推广“电子商务进社区”，启动“全国旅游刷卡无障碍桂林示范区”建设，我市列入“国家信息消费试点城市”。第三次全国经济普查及服务业统计改革试点工作取得阶段性成果。

　　(三)着力完善基础设施，城乡建设成效显著。坚持统筹规划，完善基础设施，强化环境治理，城乡面貌呈现新气象。编制完成老城23个控制性详细规划、临桂新区机场路北侧控制性详细规划和新区周边区域相关规划，县城、乡镇、村庄规划进一步完善。实施基础设施重点项目115项，完成投资105.61亿元。城镇化率达到45%。区域性交通枢纽地位凸显。湘桂铁路扩能改造工程竣工通车，我市在广西率先迈入高铁时代，连接西南、华南的贵广铁路(桂林段)建设项目完成总投资的90%；兴安至桂林高速公路竣工通车，资源至兴安高速公路开工建设，阳朔至鹿寨(桂林段)等3条续建高速公路项目累计完成年度投资计划的107%，全市从南至北的高速公路大动脉全线贯通，与周边地区高速路网的连接加快推进；两江国际机场T1航站楼改造项目开工、T2航站楼建设工程启动。临桂新区形象进一步彰显。完善交通骨架，强化基础功能，促进人气集聚，核心区实施项目86项，竣工13项，完成投资72.6亿元。临苏路等新区外联大通道建成通车，西城大道改造提升等道路桥梁项目建设加快，水电、燃气、公交、网络等基础配套设施不断完善；旅游景观水系主体工程基本完工，临桂大道展示区、行政中心区和滨水综合服务区三大节点基本成型，“一院两馆”完成竣工验收，桂林医学院临桂校区一期工程建成投入使用；金融、通信、商务、文教、医疗等一批优质资源进驻新区；大律、岩塘等7个原住民安置点加快建设。老城疏解提升迈出坚实步伐。“北通”工程西二环主体路段、福利路一期、福利路西二环立交、粟家立交与福利路连接线基本完工，滨江北路(叠彩段)、站前路、春江北路、灵川东环路基本具备通车条件，城北区域与桂兴高速、桂黄公路形成了交通流畅对接，连接新区老城的交通体系进一步完善；“南畅”工程桂林至阳朔旅游黄金大通道扩建项目实现了年内完成项目前期、年内筹资开工的目标。汽车客运北站投入使用，老城区新增和恢复停车场12个，增加停车泊位1560个，交通拥堵和停车难在一定程度上得到缓解。滨水生态广场、正阳东西巷历史文化街区和瓦窑小游园改造提升工程等项目顺利实施。强力“控违”“拆违”，控制新的违法建筑70.2万平方米，累计拆除违法建筑77.12万平方米，拆除面积比上年增加50.42万平方米，有效遏制了违法建设蔓延。推进智慧城市建设，我市成为“国家智慧城市创建试点城市”。小城镇建设力度加大。县城基础设施逐步完善，新区面积不断拓展。13个城镇化示范乡镇“书记工程”顺利推进，实施项目295个，完成投资近10亿元，基础设施和社会服务功能更加完善，产业带动和地域民族特色更加突出，一批经济繁荣、功能完善、环境舒适、宜居宜业的示范乡镇加快建设。兴坪镇、龙脊村、大塘口村、鲁家村获自治区首批特色名镇名村称号。环境综合整治纵深推进。全面落实“建设桂林国际旅游胜地要从整治环境入手”的工作要求，坚持标本兼治，强力推进环境综合整治和“美丽桂林·清洁城乡”活动，解决了一批长期积累的“老大难”问题，城市品位有力提升，城乡面貌焕然一新，全面塑造了旅游胜地新形象。城市环境综合整治“八大专项行动”投入资金1.95亿元，完成市区一批重要节点、集贸市场、垃圾中转站、公厕、停车场改造建设，市区环境卫生机械化清扫率由20%提升到60%；“美丽桂林·清洁城乡”活动多渠道筹集资金15.97亿元，环卫保洁力量进一步加强，环卫设施设备进一步完善，城乡环境卫生脏、乱、差现象大为改观，交通秩序明显改善，清洁家园、清洁水源、清洁田园取得显著成效，群众自我管理、自我监督主体意识逐步增强。创建自治区级生态乡镇8个，国家级生态村2个，自治区级生态村43个。“绿满八桂”植树造林超额完成目标任务，全市森林覆盖率达70.57%，新增城市绿化面积12.85公顷。严格落实耕地占补平衡制度，新增耕地面积1878.35公顷。对全市重点行业、企业环境风险安全隐患进行全面排查，环境监测和执法不断强化。全市每万元生产总值能耗下降3.14%，节能减排各项约束性指标完成自治区下达的目标任务。强力开展青狮潭水库环境专项整治，31万平方米的养鱼网箱全部依法拆除，水库水质明显好转，城市安全用水压力得到缓解。

　　(四)着力推进民生改善，社会事业全面进步。把保障和改善民生作为政府工作的出发点和落脚点，全市公共财政用于民生领域支出201.32亿元，占公共财政预算支出的70.43%。投入资金56.8亿元，为民办实事十项工程基本完成。社会保障水平稳步提高。推进社会保障全覆盖，各项刚性指标均完成自治区下达的年度任务。城镇新增就业7.8万人，农村劳动力转移就业9.23万人，城镇登记失业率3.76%。新农合、新农保、城镇居民养老保险参保率分别达99.16%、98.68%、98.39%；新建保障性住房9212套，完成自治区下达任务的109.7%，分配入住7136套；农村危房改造1.74万户；农村贫困人口减少7万人，贫困村人均收入增长20%。社会事业全面进步。统筹发展城乡教育，启动市区义务教育学校建设工程，进城务工人员随迁子女平等接受义务教育问题得到有效解决，学前教育三年毛入园率、义务教育巩固率、高中阶段毛入学率位居全区前列，中职教育、高等教育服务地方经济社会发展能力增强。科技创新能力持续提升，桂林国家农业科技园区、桂林国家文化和科技融合示范基地获批准建设，获得中国专利优秀奖数量及每万人口发明专利拥有量均居全区首位，成功承办第三届广西科技发明创造成果展览交易会，再获“全国科技进步先进市”。文化建设加快推进，建成170个村级公共服务中心、16个村村通广播电视乡镇无线发射台站；“百姓大舞台”获国家文化部“全国群星奖”，“百姓大讲坛”“读书月”“漓江之声”“周末大家乐”等公益性文化活动品牌深受群众欢迎，彩调《追梦》荣获“第五届中国戏剧奖·小戏小品奖”，“五通农民画引领文化致富模式”列入国家公共文化示范项目；甑皮岩正式列入第二批国家考古遗址公园名单，靖江王府及王陵国家考古遗址公园建设、灵渠世界文化遗产申报积极推进。体育事业蓬勃发展，第十二届全运会我市竞赛成绩位列全区第一。医药卫生体制改革全面推进，20类大病纳入大病保障和救助体系，新农合总额预付支付方式改革试点稳步开展。诚信计生实现全覆盖，人口计生工作保持全区领先。社会管理不断加强。“六五”普法全面开展，平安桂林建设全面推进，打击违法犯罪活动成效突出，打防管控实行一体化，“扫黄打非”取得新成效。矛盾纠纷排查化解和社会治安综合治理扎实推进，安全生产、食品药品安全等工作进一步加强，科学应对和成功处置各类突发事件，人民群众安全感和满意度不断提升。荣获“全国社会管理综合治理优秀城市”、平安中国建设最高奖“长安杯”、“全国法治城市创建先进单位”、“全国未成年人思想道德建设工作先进城市”。

　　(五)着力加强政务管理，政府服务水平有新提高。强化对《政府工作报告》目标任务落实的刚性约束和监督检查，通过细化量化，分解为287项工作指标和具体事项，全年完成276项，占总数的96.2%。建立完善重大项目领导包抓、联审联批、联席会议、“绿色通道”、“一站式”服务制度。完成“营改增”试点阶段性工作；加强政银企合作，企业及项目融资难、融资贵得到缓解。深入开展“质量强市”创建活动，评选颁发了首届“市长质量奖”。健全公众参与、专家咨询、法律审查等决策程序，自觉接受人大及其常委会的法律监督和工作监督，自觉接受政协的民主监督，人大代表建议、政协提案办结率100%、满意率均达98%以上。政务服务继续向基层延伸，清理规范行政审批事项655项，各级政务服务中心审批实际办理时限平均提速84.5%。严格执行中央“八项规定”“约法三章”等要求，市本级“三公”经费减少16.4%，市政府举行的全市性会议减少60.3%、制发的文件减少10.1%。行政权力网上公开、政务公开、政府信息公开积极推进，建立完善大督查考评机制，扎实开展纠风惠民阳光工作，廉政建设和作风建设进一步加强。

　　一年来，外事、侨务、港澳、对台、审计、物价、工商、税务、海关、检验检疫、海事、通信、邮政、供销、地震、地矿、人防、消防、保密、测绘、气象、发展研究、机关事务、地方志、社会科学、档案、宗教、老龄、残疾人等工作取得新进展，妇女儿童等事业取得新进步。国防动员和后备力量建设、双拥共建工作不断加强。民族团结进步创建活动深入开展。中直、区直驻桂林单位取得新成绩。各县区根据本地实际，发挥优势，突出特点，创新思路举措，工作亮点纷呈，县域综合经济实力显著增强，在统筹城乡经济社会发展、全面建成小康社会中发挥着十分重要的作用。全市县区和部门共获得国家级荣誉82项，自治区级荣誉120余项。

　　一年来，面对复杂环境和严峻挑战，我们践行“实干就是能力，落实才是水平”的理念，着力打造“清廉漓江、高效政府、务实桂林”，立足夯实发展基础，提升发展质量效益，营造出发展的好氛围，开创了发展的新局面，桂林的各项事业呈现出勃勃生机。这些成绩的取得，是市委总揽全局、科学决策的结果，是市人大及其常委会和市政协监督支持的结果，是全市人民群策群力、团结奋斗的结果。在此，我代表市人民政府，向全市各族人民，向驻我市中央、自治区单位，人民解放军和武警部队官兵，向各民主党派、工商联、无党派人士、人民团体及海内外所有关心支持和帮助桂林发展的各界人士，致以崇高的敬意和衷心的感谢！

　　在总结成绩的同时，我们也清醒地看到，我市经济总量不大，增长动力不足，培育新的经济增长点、推动产业结构优化升级的任务紧迫而繁重；产业规模偏小，部分骨干企业生产经营比较困难，中小企业发展环境不够宽松；政府职能转变不够，一些关系群众切身利益的问题仍然没有得到很好解决。受外需不振、内需乏力等因素影响，去年《政府工作报告》中的指标有5项增速略低于预期；因建设计划调整、重大设计变更和项目资金筹措困难等原因，6个建设项目进度偏慢或暂缓实施。我们将以对人民高度负责的态度，扎实做好工作，努力解决存在的问题，决不辜负全市人民的期望！

　　二、2014年工作安排

　　今年是深入贯彻落实党的十八届三中全会精神、全面深化改革的开局之年，是实施“十二五”规划和建设国际旅游胜地至关重要的一年。国家全面深化改革扩大开放，自治区加快打造西南中南地区新的战略支点，改革“红利”将充分释放，经济增长的内生动力活力将进一步激发；桂林国际旅游胜地建设得到了国家和自治区的高度关注和大力支持，全市一大批产业和航空、高铁、高速公路等基础设施建设的大项目好项目相继开工或竣工，桂林城市品质和发展优势将空前提升，支撑条件和发展势头将更加强劲。我们要不断地解放思想、创新理念，以全球视野拓展思维空间，按照《桂林国际旅游胜地建设发展规划纲要》和市委“加快建设新城，着力提升老城，产业融合发展，城乡协调推进，生态文化相融，富裕和谐桂林”的总要求，探索推进我市大旅游、大项目、大园区、大商业、大产业、大交通、大城建、大农镇、大研发、大文化的协同实施，进而实现我市的快发展、快提升、快变样！

　　今年政府工作的总体要求是：全面贯彻落实党的十八大和十八届二中、三中全会精神及中央和自治区党委、政府的决策部署，紧紧围绕市委四届四次全会确定的总体思路和目标任务，坚持桂林国际旅游胜地建设“一本蓝图绘到底”，坚持稳中求进工作总基调，坚持以提高发展质量和效益为中心，坚持把改革创新贯穿于经济社会发展各领域各环节，增强经济发展方式转变的内生动力，强化产业支撑、夯实发展基础、改善社会民生、优化生态环境，着力推动城市转型升级，为建成桂林国际旅游胜地、与全国全区同步全面建成小康社会打下坚实基础。

　　根据桂林市情和发展需要，今年经济社会发展的主要预期目标是：地区生产总值增长11%；组织财政收入增长11%；全社会固定资产投资增长20%；社会消费品零售总额增长16%；城镇居民人均可支配收入增长11%，农民人均纯收入增长12%；居民消费价格涨幅4%左右；城镇化率达到47.5%；万元地区生产总值能耗下降2%，主要污染物排放总量控制在自治区下达的指标之内。

　　要实现全年工作目标任务，必须抓好以下重点工作：

　　(一)发挥“龙头”带动作用，全面提速旅游胜地建设

　　紧紧围绕旅游胜地建设目标任务，抓住关键，强化措施，乘势而上，再掀旅游胜地建设新高潮。

　　强化项目带动和协调。做好规划引领、资源整合、产业衔接、板块协调和项目配套，统筹策划、布局、推进一批支撑桂林旅游转型升级的重大项目。全年实施旅游胜地建设项目495项，完成投资500亿元以上。强力推进以桂阳旅游通道为主轴的黄金旅游带、以临桂新区为核心的新城旅游区、以万达文化旅游项目为重点的文化旅游产业城、以旅游重点县为主体的旅游精品景点景区等“四大板块”旅游胜地建设。积极促进旅游项目合作，重点推进与海航联合打造航空旅游集团，设立桂林航空公司、胜地基金、胜地银行和胜地网络。

　　强化政策争取和落地。抓紧组织实施国家已批准的各项政策，跟踪落实外国游客入境免签、桂林市居民赴台自由行、境外旅客购物离境退税等获国家有关部门确认支持的政策事项，继续争取国家和自治区给予旅游胜地建设在财税、投融资、土地、交通、产业、对外开放、文化体育、机制体制等方面的政策倾斜。

　　强化旅游管理和品质提升。推进旅游管理体制改革，完善旅游发展规划。全面提升漓江风景名胜区保护、管理和营运水平，打造世界级优质旅游品牌。办好第八届联合国世界旅游组织/亚太旅游协会旅游趋势与展望国际论坛、第五届桂林国际旅游博览会。加大《中华人民共和国旅游法》实施力度，建立健全长效管理机制，实现对旅游市场秩序的全方位、全天候监管，维护良好的旅游市场秩序和公平的旅游经营环境，引导旅游企业形成可持续发展观、旅游者养成正确的旅游消费观。推进智慧旅游城市建设，建立便利的旅游公共信息平台、旅游电子商务平台和全市统一的旅游宣传平台。大力发展高端旅游，培育高铁旅游、商务旅游、生态旅游、文化旅游等新的旅游业态。大力推进旅游标准化建设，建立与国际接轨的经营管理体制，全面提升旅游服务质量和行业素质，抓好自治区特色旅游名县创建工作。力争旅游总收入增长20%以上，旅游创汇增长6%。

　　(二)抓项目促投资，增强发展驱动力

　　以重大项目建设为主战场，围绕全市发展大局，谋划及推进一批重大项目，促进投资平稳较快增长。年度实施第一批重点项目846项，完成投资752亿元以上。重点推进旅游胜地十大重中之重项目建设，确保“北通”工程全面建成、“南畅”工程全线开工，漓江保护利用和经营管理改革取得实质性成效，加快两江国际机场航站楼改扩建、中电科光通信产业园、海吉星食尚港、华润万象城和五彩城大型商业综合体、万达文化旅游产业城、保利文化产业园、体育休闲项目建设。

　　注重优化投资结构。促进更多投资投向产业、基础设施和民生领域。加快重大基础设施项目建设，年度实施铁路、公路、航空、水运等交通重点项目29项，完成投资76亿元以上；风电、水电、输变电等能源重点项目73项，完成投资39亿元以上；水库、防洪等水利重点项目25项，完成投资11亿元以上；道路桥梁、给排水等城市基础设施重点项目172项，完成投资107亿元以上。加快产业项目建设，实施产业重点项目453项，完成投资451亿元以上。加快社会公益项目建设，实施教育、文化、卫生等社会公益重点项目67项，完成投资54亿元以上。加快节能环保项目建设，实施重点项目27项，完成投资10亿元以上。强化政府性资产的调控和整合、政府投资项目资金的筹措和科学调度，构建政银合作平台，发挥各类投融资平台作用，实现新增贷款180亿元以上，争取国家核准发行企业债券10亿元。发布第四批引入民间资本项目，吸引有实力的民间资本参与投资。

　　注重项目工作统筹。全力落实各级领导联系重大项目制度，实施重大项目动态管理，推进项目协调和问题解决机制的全覆盖、高效化、常态化，完善重大项目的策划储备、联合审批、领导包抓、联席会议、跟踪服务、大督查等工作推进机制，集中力量，促进项目快开工、快建设、快竣工、快投产，使项目对全市发展的支撑更实更牢。加强项目论证，强化项目建设和资金使用的严格监管，确保项目建设安全。完善项目用地考核奖惩机制，促进集约节约用地，加大存量土地盘活力度。

　　(三)壮大增量做优存量，加快新型工业化跨越发展

　　坚持把新型工业化作为加快转变经济发展方式的主导方向和核心战略，确保更强的领导力量投入工业问题的解决，引导更多的资源整合进入工业领域，提供更优的服务促进工业政策的落实，创造更好的环境营造企业干事创业的氛围。力争规模工业增加值增长16%。

　　以企业为龙头带动产业提升。加大工业用地、项目建设、资金扶持等支持政策落实力度，建立领导联系企业“直通车”制度，严格落实工业用地占年度用地指标30%以上的政策。加大工业招大商引大资力度，扶持现有存量企业扩大规模，力争产值超50亿元企业达2家、超10亿元企业达25家、超亿元企业达430家。以龙头企业为重点，大力引进实施关联配套项目，延伸产业链条，开工建设中电科光通信产业园一期、燕京漓泉纯生易拉罐生产线技术改造等新项目，加快推进溢达九美桥时尚园、广陆数控高端数显量具和特种数控机床产业化等续建项目，完成技术改造投资增长20%以上。推动电子信息、医药及生物制品、先进装备制造、生态食品等四大优势产业集群发展，总产值占全市工业总产值比重达50%以上；积极开展创建桂林国家新型工业化(电子信息)产业示范基地工作，力争电子信息产业总产值达到360亿元。推动新材料、新能源、节能环保等战略性新兴产业加快发展，增加值占全市工业增加值比重20%以上；提高高新技术产业在规模以上工业中的比重。

　　以园区为平台促进产业集聚。创新园区管理体制机制，完善物流配送、科研创新、金融服务等生产性配套服务体系。增强高新技术产业开发区引领、示范、辐射、带动作用，推进与灵川县共建大圩产业园；开展苏桥经济开发区基础设施建设会战，加大工业产业招商引资力度，加快培育园区优势产业；突出发展西城经济开发区特色产业园区，加快建设宝山工业园；力争三大开发区工业总产值分别达310亿元、120亿元、350亿元以上。积极发展以信息服务、工业设计、服务外包等为主的都市型楼宇经济。加快县域特色工业园区建设，工业集中区工业总产值占县域规模工业比重70%以上。

　　以“两化”融合为抓手推动产业升级。拓展“两化”融合重点领域，引导鼓励企业应用信息技术改造提升制造业，积极申报国家“两化”深度融合试验区。加快“智慧城市”“宽带桂林”建设，创建国家信息消费试点城市，引导社会信息消费，培育发展物联网、云计算、大数据等新一代信息技术为支撑的新业态，推进桂林产业加快转型升级。

　　(四)夯实基础突出特色，促进农业转型升级

　　坚持把解决好“三农”问题作为重中之重，认真落实强农惠农富农政策，加大财政支农力度，全力推进“农民收入倍增计划”，不断提高农业发展质量和效益。实现农林牧渔业增加值增长5.3%。

　　做大做强特色效益农业。大力实施农户万元增收、钱粮双增工程，巩固提升6条农业万元增收示范带。实施“粮安工程”，确保粮食播种面积563万亩、总产量200万吨以上；保持水果、蔬菜、食用菌等特色农产品产量在全区的领先地位；稳定生猪、家禽生产，发展桂林特色养殖，打响桂林特色品牌，确保肉类、水产品总产量持续增长；大力发展林下经济。抓好休闲农业线路开发，发展一批惠及广大农民群众的乡村特色休闲旅游名镇、名村、星级旅游区和农家乐。加快推进桂林国家农业科技示范园区建设。建立全市性的农产品宣传、交易平台，抓好“三品一标”认证工作，健全市县乡农产品质量安全检测监管体系，加强重大动植物疫病防控，推进农产品从田头到餐桌的全过程监管。

　　推进农业农村改革。推动农村产权制度改革，发展新型农业经营方式。开展农村土地确权、登记、颁证试点工作，积极稳妥有序推进土地集中流转，鼓励土地承包经营权向农业龙头企业、专业大户、家庭农场、农民合作社流转，推动农用地“小块并大块”。深化林权制度改革，推进政策性农业保险工作。加快龙头企业集群发展，重点打造年经营收入超10亿元的大型龙头强优企业，支持海吉星、力源、康华、莱茵等大型集团加快建设农产品仓储、精深加工、流通等设施。积极发展适度规模经营，推动“一村一品“一乡一业”高效快速发展。

　　完善农业农村基础设施。桂林防洪及漓江补水枢纽工程川江水库建成蓄水、小溶江水库基本建成、斧子口水库加快续建；加快实施农田水利、水土保持、中小型病险水库除险加固、农村饮水安全等基础工程，解决30万农村人口饮水安全问题。提高农机装备水平。扎实推进新农村建设，继续实施通畅和联通工程，新增通水泥路建制村100个，新建通自然村(屯)道路200公里以上，完成1500个自然村(屯)内道路硬化，实施400个“普惠制”新村、200个自然村(屯)阳光惠农工程建设。

　　(五)拓展新业态发掘新亮点，促进现代服务业创新发展

　　坚持扩大总量与优化结构并重，继续实施服务业综合改革，推动服务业与旅游生态资源深度融合，推进服务业发展提速、比重提高、水平提升。实现第三产业增加值增长10%。

　　大力发展现代服务业。推动城市商业业态转型升级，重点发展航空经济、会展商务、现代物流、金融服务、文化创意、健康养生等现代服务业，加快打造品位休闲之都。制定全市航空经济发展规划和辐射各县的通用通勤机场布局规划，力争纳入自治区或国家行业层面航空经济规划；加快提升机场货运中转和集疏能力，做大航空旅客量；积极发展临苏经济带等区域的临空产业，扶持发展航空旅游产业，开辟市内、区内旅游航线，发展低空旅游项目，构建“旅游+航空+商业”的品质商旅模式。适应新区老城发展要求，建设功能完善、特色突出、多样化、多层次的商务中心和商业街区，重点推进万达广场、华润万象城等一批城市综合体项目建设。提升桂林会展影响力和竞争力，启动萨马兰奇文化博览园一期等高端会展项目。推进连锁经营、物流配送、电子商务等组织形式发展，积极争创国家电子商务示范城市和示范基地。大力实施“引金入桂”工程，健全多层次资本市场体系，增强金融对实体经济的服务功能。积极推进社会化养老试点，重点建设信和信智慧健康产业园、中医文化街等健康养生项目，筹办第二届健康养生服务产业创新发展高端论坛。

　　加强服务业领域改革创新。健全完善服务业重点行业发展鼓励政策，引导社会资本、生产要素投向服务业领域。大力开展服务业标准化建设，在吃、住、行、游、购、娱、会展、养老等领域建立健全服务标准体系，创建服务业知名品牌，迎接国家旅游服务业标准化试点城市评估验收。全面推进服务业统计制度改革。继续实施服务业企业培优服务工程，引进和培育竞争力强的大型集团。

　　加快培育消费新增长点。抓住桂林迎来高铁时代及建设旅游胜地的新机遇，积极培育新的都市消费新业态。继续开展“510”促销活动，大力促进旅游、汽车、健康、信息等消费，培育桂林休闲、购物、美食品牌。

　　(六)突出基础设施建设，积极推进新型城镇化

　　以人的城镇化为核心，坚持规划引领，科学设计，彰显特色，提升城镇综合承载力，打造统一与多元相结合的城市风貌，推动城乡一体化发展。

　　加快建设大交通体系。完善城市快速立体外联交通。协调推进贵广铁路(桂林段)建设，加快桂林西客站、西站东路及站前广场建设，构建与高铁车站的无缝驳接交通系统，协助做好铁路桂林动车所项目前期工作；续建资源至兴安等4条高速公路，力争灌阳(永安关)至全州(凤凰)、阳朔至鹿寨高速公路(桂林段)主体竣工，按照自治区部署力争荔浦至玉林高速公路开工，继续做好灌阳经恭城至平乐高速公路招商工作；协调推进两江国际机场项目建设，创建国际卫生机场；继续推进磨盘山客运港、平乐印山旅游码头等西江黄金水道项目建设。完善市区路网结构。围绕实施“北通、南畅、东拓、西联”战略，以桂黄公路和桂阳公路为主轴，加快推进福利路二期、西二环北辰立交、灵川西环路、雁山科教组团中心环路建设，实现南北大通道的全线立体对接；强力推进万福东路、龙门大桥、中隐路西延长线、芳香东路等项目建设，打造老城和新区东西连接的快速通道；强力推进临桂新区新中北路、万南路、环西路桥等20项道路桥梁项目建设，实现新区纵横路网全面连接贯通；建立老城通往临桂、灵川的城市大公交体系，开工建设汽车南站，完成汽车东站前期工作，创建“公交都市”示范城市。

　　加快中心城区建设。围绕临桂新区满足进驻和入住条件，进一步完善临桂新区周边区域的相关规划，强力推进保利文化艺术中心、怡化体育休闲小镇等重点招商项目建设，加快新区物流配套设施建设；继续充实临桂大道展示区、创业大厦行政中心区和滨水综合服务区三大节点，尽快形成金融、商贸、美食文化等特色街区；增强新区生活服务配套能力，加快推进广电中心、桂林中学临桂校区、临桂新区综合医院等项目建设。围绕老城疏解改造提升，进一步完善功能、突出特色、彰显文旅、提升品位、优化环境。重点推进棚户区改造，启动秀峰区甲山、象山区田心村、七星区下关村、叠彩区福利路、雁山区雁山镇等第一批12个棚户区改造项目；加快推进滨水生态广场、正阳路东西巷、桃花湾特色景区、大河圩村、瓦窑片区、福隆园、草坪老街等重要节点、城中村综合改造；充分挖掘历史文化内涵，加强城市建筑特色规划设计，完善国际旅游城市标识导览系统，积极推广绿色建筑，打造城市景观，改善人居环境，建设地域特征鲜明、历史文化名城气韵充沛的特色示范小区。

　　精心培育县城和小城镇。制定实施桂林新型城镇化发展规划，修编完善桂林城镇体系规划，强力推进村镇规划集中行动，构建合理的城镇体系和可持续发展的空间格局。实施大县城战略，鼓励各县按照规划定位，实行差异化发展，优化县城空间结构，强化承载功能、产业功能、服务功能和集聚功能，形成鲜明的地域优势和产业优势，辐射带动县域小城镇发展。有重点地发展小城镇，推动乡镇所在地市政基础设施向周边农村延伸、公共服务向农村覆盖；强力推进13个城镇化示范乡镇“书记工程”，打造经济强镇、产业强镇、旅游强镇、特色强镇。

　　(七)深化重点领域改革，增强发展动力活力

　　积极推动改革创新，破解发展难题，增创发展优势，充分释放市场潜能和改革“红利”。

　　深化经济体制改革。实施财源培植目标责任制和考评奖惩制度，加强政府债务、政府项目资金及其他财政资金的严格统一监管，确保财经纪律落实到位。深化财政预算管理和国库管理改革，创新财政资金扶持企业发展方式，增强财政资金的引导和放大作用。完善国有资产管理体制，改革国有资本经营体制。加大国有企业改革重组力度，支持和鼓励国有资本投资项目吸收非国有资本参股，发展混合所有制经济。引导和鼓励发展新型城市工业。深化土地管理制度改革，落实旅游产业用地试点政策。做好第三次全国经济普查相关工作，创新经济社会发展动态管理模式，探索建立产业、生态、县区发展的差异化科学评价体系。

　　加快推进社会领域改革。进一步理顺政府和社会的关系，推进社会事业“管办分离”，做到“管”得更科学公正，“办”得更规范高效。创新公共服务供给模式，大力推进环卫作业、市政设施和园林养护等领域向社会购买服务。创新社会治理方式，推进基层民主自治，整合城乡基层设立的各种工作机构，加强乡镇(街道)社会管理服务中心和社区综合管理服务站建设，充分发挥行业协会、商会、慈善组织、义工志愿者组织等各类社会组织的桥梁纽带作用。

　　实施科技创新驱动战略。建立健全政府引导、市场导向、研发应用、产权保护相结合的科技创新发展体系，加强工程技术研究中心、科技示范园区等载体建设，加快建设桂林国家文化和科技融合示范基地，完善科技孵化机制，建立完善科技创新服务平台。积极培育企业创新主体，营造科技型中小企业创新和高层次科技人才创业的良好环境，重点打造战略性新兴产业创新集群。深入实施发明专利倍增计划，力争每万人口发明专利拥有量2.5件。继续开展“质量强市”创建工作。

　　大力发展非公有制经济。消除各种隐形壁垒，确保非公有制资本享有平等的市场准入，从金融、税收、信用担保等方面完善支持非公有制企业做大做强的政策，完善支持民间投资健康发展的配套措施。鼓励引导和推动非公有制企业引入各类资本优化产权结构，建立现代企业制度，完善企业自律机制。完善中小微企业服务体系，创新投资、担保、贷款联动的融资方式，适时设立专项发展基金。

　　统筹城乡一体化发展。推进城乡基本公共服务均等化，在全市逐步建立起多层级的公共服务网络体系，实现进城农民工、失地农民与市民基本公共服务和公共产品的均等化，把进城落户农民逐步纳入城镇住房和社会保障体系。把发展壮大县域经济作为促进全市城乡统筹发展的重要任务，因地制宜，突出特色，创造和发挥比较优势，合理选择和大力培育特色优势主导产业，强化县域经济发展的产业支撑，充分激发县域经济发展的动力和活力。

　　实现开放型经济新发展。加强与珠三角、湘粤黔区域以旅游为重点的全方位合作。着力引进优质企业和大项目，加强在谈、签约、在建重大引资项目对接落实和跟踪服务，支持我市企业参与境外资源开发和经济合作。抓好外贸转型基地和转型示范企业建设。力争招商引资实际到位资金超600亿元，实际利用外资超4亿美元，进出口贸易总额增长5%。

　　(八)推进生态文明建设，打造美丽桂林

　　深化城乡环境综合整治。坚持高标准规划、高质量建设、高水平管理，推进城市管理机制创新，真正做到“规划一张图、审批一支笔、建设一盘棋、管理一条龙”。强力推进“控违”“拆违”专项整治行动，力争新的违法建设“零增长、全拆除、不反弹”、存量违法建筑逐步拆除。按照“城市面貌发生根本性变化、城市管理水平达到全国先进行列”的目标要求，整合规划、住建、市政、市容、园林、环保等城市管理相关部门职能，完善综合执法体制，继续推进环境综合整治“八大专项行动”，重拳治堵、重点治脏、切实治乱、全面治差、彻底治本，切实巩固市容环境综合整治成果。推进城乡生活污水和垃圾处理系统配套建设，完善垃圾分类、资源化利用，进一步提高生活垃圾无害化处理率。加强农村环境综合整治，建立“美丽桂林·清洁城乡”活动长效机制，启动生态乡村活动，实施村屯绿化、村庄道路硬化、水源净化工程，提升城乡环境卫生精细化管理水平。提高城市文明综合指数，争创全国文明城市。

　　加强环境治理和生态保护。全面加大环境监测和执法力度，抓好污染源头与环境隐患排查治理，严格落实环保目标责任制和环保“一票否决制”，统筹推进治水、治山、治大气。全面实施《广西壮族自治区漓江流域生态环境保护条例》及《桂林漓江风景名胜区总体规划》，编制控制性详细规划，抓好环境重点整治，加强漓江生态环境保护，实施漓江小流域专项整治，推动建立漓江保护生态补偿机制。加强饮用水源保护，全面完成青狮潭水库水质环境综合治理，建立长效管理机制，推进城市备用水源建设。继续开展“绿满八桂”植树造林活动，积极创建国家生态园林城市。落实最严格的耕地保护制度和节约集约用地制度，加强地质灾害、矿山地质环境治理，加快永久基本农田划定。加强农村环境连片整治，防止农业面源污染。强化大气污染防控，整治扬尘、机动车尾气等污染，努力提高市区环境空气质量优良天数。

　　推进节能减排。扎实开展低碳城市试点工作，加强节能减排公共检测平台建设，编制碳清单，建立碳排放核算体系。积极推广节能技术应用，加快太阳能、风能等可再生清洁能源产业发展和应用，加快推进建筑节能和公共节能，开展临桂新区绿色生态城区建设。深入开展国家“万家企业节能低碳行动”，加强合同能源管理，切实抓好41家重点企业耗能节能目标考核。抓好循环经济示范企业建设和示范园区改造。良好的生态环境是桂林的最大优势和最大特色，我们要切实走出一条有别于传统发展方式的新路子，在促进经济不断发展的同时，让桂林的山更青、水更绿、天更蓝、空气更清新。

　　(九)突出民生优先，加快发展社会事业

　　今年用于民生领域的公共财政支出占公共财政预算支出比重达到70%以上，着力解决就学、就业、就医、社保等重点民生问题，让发展成果更多惠及百姓。

　　全面推进城乡统筹就业。完善城乡均等的公共就业创业服务体系，加强市、县区、乡镇公共就业服务网络平台建设，实施高校毕业生创业就业补贴，加大公益性岗位开发与职业技能培训力度，实现城镇新增就业6.8万人，城镇失业人员再就业1.5万人，城镇失业率控制在4.5%以内。

　　健全社会保障体系。整合城乡居民养老保险和基本医疗保险制度，加强社保基金征缴和监管力度，新农保和城居保参保率完成自治区下达任务；加快新农合支付方式改革，确保新农合参合率达95%以上；提高城乡低保、农村五保供养、灾害救助等社会救助水平和保障标准；健全社会养老服务体系，年内新建居家养老服务中心10个，满足5万名社会老人养老需求；新建保障性住房6690套，分配入住6000套，实施农村危房改造2.02万户。

　　扎实开展扶贫攻坚。全面启动第二批138个整村推进贫困村的扶贫攻坚战，加快贫困地区脱贫致富步伐，实现农村贫困人口减少12%。

　　办好人民满意教育。全面深化教育领域综合改革，提高教育质量，促进教育公平。实施以扩容提质增速为主要内容的教育发展十大重点工程。建立市教育发展基金，完善和落实覆盖全学段的家庭经济困难学生资助政策。推进学前、九年义务、普通高中阶段教育，完成中职教育归口管理，积极支持高校发展。健全中小学安全工作长效机制，维护好学校安全。

　　加快发展文化事业和文化产业。深化文化体制改革，激发文化创造活力，强化文化服务功能，不断满足广大市民和游客的文化需求。加强城乡公共文化基础设施建设，新建150个村级公共服务中心，进一步推进公共文化设施免费开放，完成村村通广播电视乡镇无线发射台站建设任务。强化文艺精品创作，培育地方文化品牌，提升“百姓大舞台”“百姓大讲坛”“读书月”“漓江之声”“周末大家乐”等群众文化品牌影响力。大力推进金世邦国际足球旅游文化产业园、“夜王城”大型演出等重大文化产业项目建设，继续抓好灵渠申报世界文化遗产工作，加强靖江王府及王陵、甑皮岩国家考古遗址公园的建设、保护和利用。开展桂林历史文化遗存及文化元素的调查、挖掘、整理、保存和利用，使桂林历史文化名城的魅力更加彰显。完善文化产业发展激励机制，培育壮大文化市场主体，加快发展创意、数字出版、动漫游戏等新型文化业态，促进演艺、娱乐、网络、艺术品、出版物市场等产业快速健康发展。广泛开展全民健身运动，努力提高体育竞技水平，加快罗山湖体育运动休闲项目建设，稳步推进体育休闲产业发展。

　　加快发展医疗卫生事业。实施卫生强基、人才培养、中医提升三大工程，加强卫生信息化建设。抓好国家卫生城市复审工作。推进县级公立医院改革，巩固完善基本药物制度和基层运行新机制，稳步提升公共卫生服务水平。抓好门诊统筹和大病医疗保障试点工作，健全覆盖城乡的医疗急救服务体系。有效防控艾滋病等重大疾病。根据国家部署调整完善计划生育政策，抓好流动人口卫生和计划生育基本公共服务均等化试点工作，继续做好诚信计生巩固提质工作。

　　健全公共安全体系。深入实施“六五”普法，健全社区矫正制度。开展社会矛盾纠纷排查化解工作，完善社会治安打防管控一体化建设，推进平安桂林建设。加强“扫黄打非”工作，净化社会文化环境。切实抓好安全生产工作，强化对事故灾难、公共卫生、食品药品安全、环境安全事件的预防预警、督促检查和应急处置。全面做好国防动员、民兵预备役和双拥共建工作，深入推进军民融合发展。

　　实施为民办实事工程。察民情、听民声、知民意，及时回应社会诉求，建立政府主导的解决人民群众“眼前困难身边事”的工作机制，围绕人民群众反映强烈、迫切需要解决的热点难点问题，办实事、解难题、惠民生。坚持民生优先、急需先行，多渠道筹措资金，实施社保、健康、教育、强基、安居、土地整治、农补、生态、文化、交通等10项为民办实事工程，同时，从今年开始，每年解决一批事关城乡居民生产生活环境方面的眼前困难身边事。

　　三、加快转变政府职能，努力优化发展环境

　　完成今年经济社会发展目标任务，关键要加强政府自身的改革和建设。各级政府、政府各部门和政府工作人员要以深入开展党的群众路线教育实践活动为动力，按照为民、务实、清廉的要求，坚决反对“四风”，整治机关“六病”等突出问题，更加注重做好打基础利长远的工作，更加注重做好建机制利长效的工作，更加注重做好涉及人民群众衣食住行的工作。以“立即执行”理念全力推进各项工作的最快落实，以“知难而进”理念大力推进大发展大建设，以“敢于亮剑”理念强力推进改革创新，以“用心做事”理念着力推进民生改善，以“持续学习”理念倾力推进现代政府文化体系的形成，以“继承创新”理念奋力推进政府管理体系的创新，努力构建集学习政府、改革政府、服务政府、高效政府、责任政府、法治政府为一体的政府运行模式。

　　把依法行政作为政府工作的根本要求，严格依照法定权限和法定程序行使权力、履行职责。自觉接受市人大及其常委会的监督，政府重大决策出台前向人大报告；自觉接受市政协的民主监督，认真听取各民主党派、工商联、无党派人士和人民团体的意见。坚持科学决策、民主决策、依法决策，强化重大决策跟踪反馈和责任追究制度。充分发挥市场在资源配置中的决定性作用，继续推进政企、政事、政社分开，加快行政审批制度改革，进一步简政放权，市场机制能有效调节的经济活动一律取消审批，直接面向社会的具体服务管理事项原则上下放县区实施，把政府工作重点转到创造良好发展环境、提供优质公共服务和维护社会公平正义上来。各级政府和政府各部门要主动负责，认真履责，敢于担责。建立横向到边、纵向到底、责任到人、责任到事的工作推进机制和行政问责机制，确保政令畅通。

　　切实加强政府廉政建设。严格执行“八项规定”和“约法三章”，全面落实党政机关厉行节约反对浪费条例等各项政策规定，严格控制“三公”经费。深入推进惩治和预防腐败体系建设，强化对重点领域、关键环节、重要岗位权力运行的监督制约，严肃查处各类违法违纪案件。推进政务公开和政府信息公开，探索推行各级政府及工作部门权力清单制度。做好行政监察和审计监督，重视司法、舆论、公众监督，运用和规范互联网监督，让政府工作置于全方位监督之下。加强政风行风建设，扩大投资环境和纠风工作评价的群众参与度，坚决纠正损害群众利益的不正之风。

　　各位代表，桂林发展，我们重任在肩，人民福祉，我们责无旁贷！让我们紧密团结在以习近平同志为总书记的党中央周围，高举中国特色社会主义伟大旗帜，以邓小平理论、“三个代表”重要思想、科学发展观为指导，在市委的领导下，齐心协力，开拓创新，扎实工作，为加快建成桂林国际旅游胜地、全面建成小康社会而努力奋斗！
